# 2-(4-Methyl­phen­oxy)-5-nitro­pyridine

**DOI:** 10.1107/S1600536811044047

**Published:** 2011-10-29

**Authors:** Shah Bakhtiar Nasir, Zainal Abidin Fairuz, Zanariah Abdullah, Seik Weng Ng, Edward R. T. Tiekink

**Affiliations:** a Department of Chemistry, University of Malaya, 50603 Kuala Lumpur, Malaysia; b Chemistry Department, Faculty of, Science, King Abdulaziz University, PO Box 80203 Jeddah, Saudi Arabia

## Abstract

The title mol­ecule, C_12_H_10_N_2_O_3_, is twisted, the dihedral angle between the rings being 61.16 (13)°. The nitro group is approximately coplanar with the pyridine ring to which it is attached [O—N—C—C torsion angle = −178.1 (3)°]. Supra­molecular chains along [010] and mediated by C—H⋯O and π–π [centroid(pyrid­yl)–(benzene) distance = 3.8259 (18) Å] contacts feature in the crystal packing.

## Related literature

For the structure of a related nitro-pyridine derivative, see: Nasir *et al.* (2010 ▶).
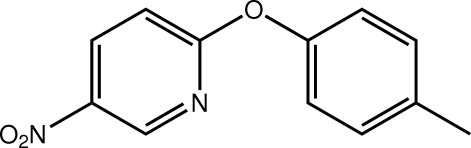


## Experimental

### 

#### Crystal data

C_12_H_10_N_2_O_3_
*M* *_r_* = 230.22
Orthorhombic, 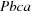

*a* = 7.2818 (18) Å
*b* = 11.977 (2) Å
*c* = 25.362 (5) Å
*V* = 2211.9 (8) Å^3^
*Z* = 8
Mo *K*α radiation
μ = 0.10 mm^−1^
*T* = 293 K
0.20 × 0.18 × 0.07 mm

#### Data collection

Bruker SMART APEX diffractometer
Absorption correction: multi-scan (*SADABS*; Sheldrick, 1996 ▶) *T*_min_ = 0.670, *T*_max_ = 0.746
15887 measured reflections
1951 independent reflections
1089 reflections with *I* > 2σ(*I*)
*R*_int_ = 0.074

#### Refinement

*R*[*F* ^2^ > 2σ(*F* ^2^)] = 0.047
*wR*(*F* ^2^) = 0.147
*S* = 1.02
1951 reflections
156 parameters
H-atom parameters constrained
Δρ_max_ = 0.15 e Å^−3^
Δρ_min_ = −0.13 e Å^−3^

### 

Data collection: *APEX2* (Bruker, 2009 ▶); cell refinement: *SAINT* (Bruker, 2009 ▶); data reduction: *SAINT*; program(s) used to solve structure: *SHELXS97* (Sheldrick, 2008 ▶); program(s) used to refine structure: *SHELXL97* (Sheldrick, 2008 ▶); molecular graphics: *ORTEP-3* (Farrugia, 1997 ▶) and *DIAMOND* (Brandenburg, 2006 ▶); software used to prepare material for publication: *publCIF* (Westrip, 2010 ▶).

## Supplementary Material

Crystal structure: contains datablock(s) global, I. DOI: 10.1107/S1600536811044047/hg5116sup1.cif

Structure factors: contains datablock(s) I. DOI: 10.1107/S1600536811044047/hg5116Isup2.hkl

Supplementary material file. DOI: 10.1107/S1600536811044047/hg5116Isup3.cml

Additional supplementary materials:  crystallographic information; 3D view; checkCIF report

## Figures and Tables

**Table 1:** Hydrogen-bond geometry (Å, °)

*D*—H⋯*A*	*D*—H	H⋯*A*	*D*⋯*A*	*D*—H⋯*A*
C5—H5⋯O(3)^i^	0.93	2.43	3.135 (3)	132

Symmetry code: (i) 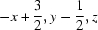.

**Fig. 1.**
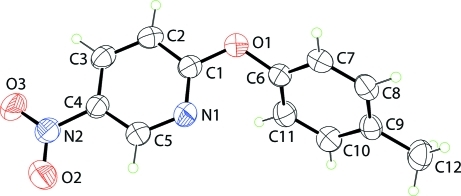
The molecular structure of (I) showing the atom-labelling scheme and displacement ellipsoids at the 35% probability level.

**Fig. 2.**
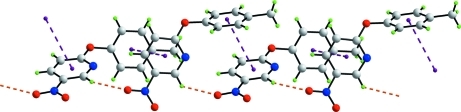
Supramolecular chain along [010] in (I) sustained by C—H···O and π–π interactions, shown as orange and purple dashed lines, respectively.

**Fig. 3.**
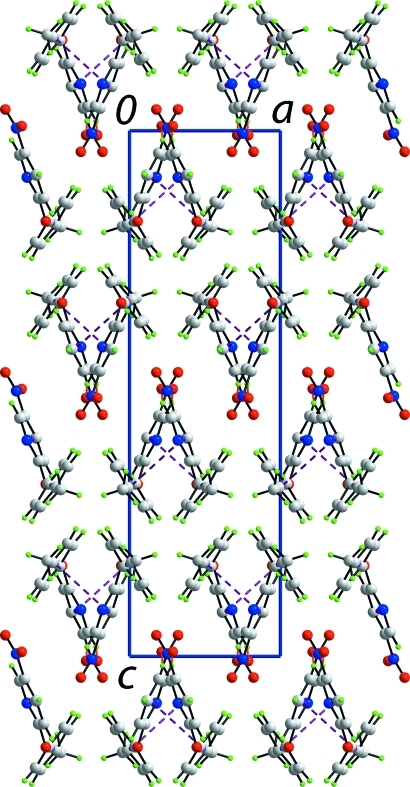
Unit-cell contents for (I) shown in projection down the b axis highlighting the packing of supramolecular chains.

## supplementary crystallographic information

### Comment

The synthesis and crystal structure determination of the title compound, (I), was determined in connection with studies of related species (Nasir *et al.*, 2010). In (I), the dihedral angle formed between the pyridyl and benzene rings is 61.16 (13)°, indicating significant twisting in the molecule. The nitro group is co-planar with the pyridyl ring to which it is connected as seen in the value of the O2—N2—C4—C3 torsion angle of -178.1 (3)°.

The most prominent features in the crystal packing are the formation of C—H···O, Table 1, and π–π interactions. The latter occur between the pyridyl and benzene rings with the separation between the ring centroids being 3.8259 (18) Å for symmetry operation 3/2 - *x*, 1/2 + *y*, *z*. These interactions lead to supramolecular chains along the *b* axis, Fig. 2, which pack as shown in Fig. 3.

### Experimental

*p*-Cresol (2.16 g, 20 mmol) and sodium hydroxide (0.80 g, 20 mmol) were dissolved in water (50 ml) and to the solution was added 2-chloro-5-nitropyridine (3.17 g, 20 mmol) dissolved in THF (50 ml). The mixture was heated for 5 h. Water was added and the organic phase extracted with chloroform. The chloroform solution was dried over sodium sulfate; slow evaporation led to the formation of colourless crystals.

### Refinement

Hydrogen atoms were placed at calculated positions (C—H 0.93–0.96 Å) and were treated as riding on their parent carbon atoms, with *U*(H) set to 1.2–1.5*U*_eq_(C).

### Crystal data

**Table:**

C_12_H_10_N_2_O_3_	*F*(000) = 960
*M**_r_* = 230.22	*D*_x_ = 1.383 Mg m^−^^3^
Orthorhombic, *P**b**c**a*	Mo *K*α radiation, λ = 0.71073 Å
Hall symbol: -P 2ac 2ab	Cell parameters from 1038 reflections
*a* = 7.2818 (18) Å	θ = 3.2–19.7°
*b* = 11.977 (2) Å	µ = 0.10 mm^−^^1^
*c* = 25.362 (5) Å	*T* = 293 K
*V* = 2211.9 (8) Å^3^	Block, colourless
*Z* = 8	0.20 × 0.18 × 0.07 mm

### Data collection

**Table:**

Bruker SMART APEX diffractometer	1951 independent reflections
Radiation source: fine-focus sealed tube	1089 reflections with *I* > 2σ(*I*)
graphite	*R*_int_ = 0.074
ω scans	θ_max_ = 25.0°, θ_min_ = 1.6°
Absorption correction: multi-scan (*SADABS*; Sheldrick, 1996)	*h* = −8→8
*T*_min_ = 0.670, *T*_max_ = 0.746	*k* = −14→14
15887 measured reflections	*l* = −30→30

### Refinement

**Table:**

Refinement on *F*^2^	Secondary atom site location: difference Fourier map
Least-squares matrix: full	Hydrogen site location: inferred from neighbouring sites
*R*[*F*^2^ > 2σ(*F*^2^)] = 0.047	H-atom parameters constrained
*wR*(*F*^2^) = 0.147	*w* = 1/[σ^2^(*F*_o_^2^) + (0.0643*P*)^2^ + 0.2807*P*] where *P* = (*F*_o_^2^ + 2*F*_c_^2^)/3
*S* = 1.02	(Δ/σ)_max_ = 0.001
1951 reflections	Δρ_max_ = 0.15 e Å^−^^3^
156 parameters	Δρ_min_ = −0.13 e Å^−^^3^
0 restraints	Extinction correction: *SHELXL97* (Sheldrick, 2008), Fc^*^=kFc[1+0.001xFc^2^λ^3^/sin(2θ)]^-1/4^
Primary atom site location: structure-invariant direct methods	Extinction coefficient: 0.0060 (13)

### Fractional atomic coordinates and isotropic or equivalent isotropic displacement parameters (Å^2^)

**Table:**

	*x*	*y*	*z*	*U*_iso_*/*U*_eq_	
O1	0.5542 (3)	0.41426 (14)	0.32750 (7)	0.0725 (6)	
O2	0.8217 (3)	0.59307 (19)	0.54024 (9)	0.1039 (8)	
O3	0.7135 (4)	0.74378 (19)	0.50863 (8)	0.1135 (9)	
N1	0.6779 (3)	0.41528 (17)	0.41124 (9)	0.0699 (7)	
N2	0.7486 (4)	0.6456 (2)	0.50490 (10)	0.0758 (7)	
C1	0.6005 (4)	0.4707 (2)	0.37191 (11)	0.0595 (7)	
C2	0.5682 (3)	0.5841 (2)	0.37215 (10)	0.0613 (7)	
H2	0.5145	0.6191	0.3433	0.074*	
C3	0.6168 (4)	0.6439 (2)	0.41563 (10)	0.0648 (7)	
H3	0.5978	0.7206	0.4173	0.078*	
C4	0.6950 (4)	0.5868 (2)	0.45701 (10)	0.0576 (7)	
C5	0.7247 (4)	0.4751 (2)	0.45359 (11)	0.0675 (8)	
H5	0.7798	0.4389	0.4819	0.081*	
C6	0.5381 (4)	0.2976 (2)	0.32825 (10)	0.0588 (7)	
C7	0.6205 (4)	0.2394 (2)	0.28825 (9)	0.0625 (7)	
H7	0.6943	0.2759	0.2638	0.075*	
C8	0.5920 (4)	0.1252 (2)	0.28494 (10)	0.0639 (7)	
H8	0.6459	0.0857	0.2574	0.077*	
C9	0.4864 (4)	0.0685 (2)	0.32118 (10)	0.0601 (7)	
C10	0.4088 (4)	0.1301 (2)	0.36136 (11)	0.0675 (8)	
H10	0.3393	0.0936	0.3868	0.081*	
C11	0.4313 (4)	0.2442 (2)	0.36485 (10)	0.0685 (8)	
H11	0.3747	0.2844	0.3917	0.082*	
C12	0.4559 (4)	−0.0556 (2)	0.31771 (12)	0.0854 (9)	
H12A	0.4765	−0.0888	0.3517	0.128*	
H12B	0.5397	−0.0871	0.2926	0.128*	
H12C	0.3320	−0.0700	0.3067	0.128*	

### Atomic displacement parameters (Å^2^)

**Table:**

	*U*^11^	*U*^22^	*U*^33^	*U*^12^	*U*^13^	*U*^23^
O1	0.0976 (16)	0.0589 (12)	0.0612 (11)	0.0000 (10)	−0.0111 (11)	0.0016 (9)
O2	0.142 (2)	0.0885 (16)	0.0806 (14)	0.0159 (14)	−0.0259 (15)	−0.0119 (13)
O3	0.210 (3)	0.0523 (12)	0.0784 (14)	−0.0020 (15)	0.0069 (16)	−0.0057 (11)
N1	0.0881 (17)	0.0516 (13)	0.0699 (14)	0.0073 (11)	−0.0110 (14)	−0.0033 (12)
N2	0.104 (2)	0.0597 (16)	0.0639 (15)	−0.0023 (14)	0.0124 (14)	−0.0004 (13)
C1	0.0622 (17)	0.0557 (16)	0.0607 (16)	0.0015 (13)	0.0020 (14)	0.0036 (14)
C2	0.0640 (18)	0.0586 (17)	0.0615 (16)	0.0076 (13)	0.0040 (14)	0.0104 (14)
C3	0.0754 (19)	0.0496 (15)	0.0695 (17)	0.0059 (14)	0.0120 (16)	0.0042 (14)
C4	0.0645 (17)	0.0508 (16)	0.0574 (15)	−0.0002 (13)	0.0085 (14)	−0.0002 (13)
C5	0.081 (2)	0.0566 (17)	0.0650 (17)	0.0056 (15)	−0.0076 (15)	0.0019 (14)
C6	0.0642 (18)	0.0568 (17)	0.0555 (16)	0.0006 (13)	−0.0087 (14)	−0.0002 (13)
C7	0.0632 (17)	0.0729 (18)	0.0513 (15)	−0.0010 (14)	−0.0007 (13)	0.0029 (14)
C8	0.0646 (17)	0.0730 (19)	0.0541 (15)	0.0055 (15)	−0.0050 (14)	−0.0100 (14)
C9	0.0536 (16)	0.0654 (18)	0.0614 (16)	−0.0024 (14)	−0.0105 (14)	−0.0089 (14)
C10	0.0596 (17)	0.075 (2)	0.0683 (18)	−0.0082 (14)	0.0032 (15)	0.0007 (15)
C11	0.0697 (19)	0.0705 (19)	0.0651 (17)	0.0041 (15)	0.0042 (15)	−0.0086 (15)
C12	0.088 (2)	0.071 (2)	0.098 (2)	−0.0109 (16)	−0.0140 (19)	−0.0137 (17)

### Geometric parameters (Å, °)

**Table:**

O1—C1	1.356 (3)	C6—C11	1.369 (3)
O1—C6	1.403 (3)	C6—C7	1.369 (3)
O2—N2	1.217 (3)	C7—C8	1.386 (4)
O3—N2	1.208 (3)	C7—H7	0.9300
N1—C1	1.324 (3)	C8—C9	1.377 (3)
N1—C5	1.336 (3)	C8—H8	0.9300
N2—C4	1.457 (3)	C9—C10	1.379 (3)
C1—C2	1.379 (3)	C9—C12	1.505 (3)
C2—C3	1.361 (3)	C10—C11	1.380 (3)
C2—H2	0.9300	C10—H10	0.9300
C3—C4	1.376 (3)	C11—H11	0.9300
C3—H3	0.9300	C12—H12A	0.9600
C4—C5	1.358 (3)	C12—H12B	0.9600
C5—H5	0.9300	C12—H12C	0.9600
			
C1—O1—C6	120.4 (2)	C7—C6—O1	117.4 (2)
C1—N1—C5	116.4 (2)	C6—C7—C8	118.8 (3)
O3—N2—O2	122.5 (3)	C6—C7—H7	120.6
O3—N2—C4	118.6 (3)	C8—C7—H7	120.6
O2—N2—C4	118.8 (2)	C9—C8—C7	122.0 (2)
N1—C1—O1	118.8 (2)	C9—C8—H8	119.0
N1—C1—C2	124.3 (3)	C7—C8—H8	119.0
O1—C1—C2	116.9 (2)	C8—C9—C10	117.3 (2)
C3—C2—C1	118.5 (2)	C8—C9—C12	122.0 (2)
C3—C2—H2	120.7	C10—C9—C12	120.7 (3)
C1—C2—H2	120.7	C9—C10—C11	121.9 (3)
C2—C3—C4	117.7 (2)	C9—C10—H10	119.0
C2—C3—H3	121.2	C11—C10—H10	119.0
C4—C3—H3	121.2	C6—C11—C10	119.1 (3)
C5—C4—C3	120.4 (3)	C6—C11—H11	120.4
C5—C4—N2	119.1 (3)	C10—C11—H11	120.4
C3—C4—N2	120.5 (2)	C9—C12—H12A	109.5
N1—C5—C4	122.7 (3)	C9—C12—H12B	109.5
N1—C5—H5	118.7	H12A—C12—H12B	109.5
C4—C5—H5	118.7	C9—C12—H12C	109.5
C11—C6—C7	120.9 (2)	H12A—C12—H12C	109.5
C11—C6—O1	121.5 (2)	H12B—C12—H12C	109.5
			
C5—N1—C1—O1	178.5 (2)	C3—C4—C5—N1	−1.2 (4)
C5—N1—C1—C2	0.7 (4)	N2—C4—C5—N1	179.4 (2)
C6—O1—C1—N1	18.3 (4)	C1—O1—C6—C11	52.0 (3)
C6—O1—C1—C2	−163.8 (2)	C1—O1—C6—C7	−133.5 (2)
N1—C1—C2—C3	−0.7 (4)	C11—C6—C7—C8	0.9 (4)
O1—C1—C2—C3	−178.6 (2)	O1—C6—C7—C8	−173.7 (2)
C1—C2—C3—C4	−0.2 (4)	C6—C7—C8—C9	−1.2 (4)
C2—C3—C4—C5	1.1 (4)	C7—C8—C9—C10	0.0 (4)
C2—C3—C4—N2	−179.4 (2)	C7—C8—C9—C12	−179.8 (2)
O3—N2—C4—C5	−176.4 (3)	C8—C9—C10—C11	1.6 (4)
O2—N2—C4—C5	1.3 (4)	C12—C9—C10—C11	−178.6 (3)
O3—N2—C4—C3	4.1 (4)	C7—C6—C11—C10	0.7 (4)
O2—N2—C4—C3	−178.1 (3)	O1—C6—C11—C10	175.0 (2)
C1—N1—C5—C4	0.2 (4)	C9—C10—C11—C6	−1.9 (4)

### Hydrogen-bond geometry (Å, °)

**Table:**

*D*—H···*A*	*D*—H	H···*A*	*D*···*A*	*D*—H···*A*
C5—H5···O(3)^i^	0.93	2.43	3.135 (3)	132

Symmetry codes: (i) −*x*+3/2, *y*−1/2, *z*.
